# Observations on the Emmenagogue Properties of Polygala Senega

**Published:** 1850-11

**Authors:** Casper Morris

**Affiliations:** Philadelphia


*Observations on the Emmenagogue properties of Polygala Senega.* By CASPER MORRIS, M. D., of Philadelphia.—Among the articles contributed to the materia medica by our own country, not one is more important than the polygala senega. However little its virtues may be esteemed abroad, there are few American physicians who do not recognize its importance in the treatment of certain stages of croup and bronchitis. My present object, however, is not to celebrate its praises in affections in which its value is so generally appreciated, but to draw attention to its effects in a class of cases which often baffle the efforts of the physician, and cause no little anxiety to the patient; to properties which, though recognized before, have been overlooked or forgotten. It is now more than twenty years since my attention was first directed to the emmenagogue properties of this root. I cannot recall the source from which the knowledge of its virtues was derived, but am disposed to ascribe it to the teaching of Professor Chapman, as I find, on reference to his work on therapeutics, that he speaks of them in very strong terms of commendation, and gives the credit of first drawing the attention of the profession to them, to the late Dr. Joseph Hartshorne. At the period to which I refer, I was induced to direct the employment of the senega for an unmarried lady, of about thirty years of age, suffering from suppression of the menstrual discharge of several months duration, combined with a catarrhal affection. So prompt was the restoration of the uterine discharge, that I considered it a mere coincidence, and remarked it as one of those cases of facts which may be misapplied so as to teach error instead of truth. Since then I have had ample opportunity to verify its claims to the credit of the result.

The tendency of its influence to the sexual and urinary organs has often since arrested my attention, in cases of children to whom I have given it for croup, in which I have found difficult micturition follow its use, sometimes to a degree quite inconvenient. Pereira mentions, among its physiological effects, "increased secretion of urine, and a feeling of heat in the urinary passages," and adds: "it appears to excite moderately the vascular system, to promote the secretions (at least those of the kidneys and skin, *uterus* and bronchial membrane), and to exert a specific influence over the nervous system;" he mentions the fact that it has been used as an emmenagogue in amenorrhœa." In the Dispensatory of Wood and Bache, there is a mere casual allusion to its having been recommended in amenorrhæa; while Dr. Eberle refuses credence to the assertion that it possesses any emmenagogue properties. The strong testimony of Dr. Chapman deserves to be disseminated anew, as it may be overlooked among the many modern works on materia medica and pharmacy. I shall therefore furnish it for the benefit of your readers.

He introduces it first on the list of emmenagogues in the following terms;

"To Dr. Hartshorne, of this city, we owe the credit of having discovered the properties of this article as an emmenagogue. Conversing with him, some years ago, on the difficulty of managing certain forms of amenorrhœa, by the common treatment, he told me that he thought he had used it with advantage in these cases. Confiding in the accuracy of his observations, I determined to lose no time in making trial of the medicine. This I have done since, both in my public and private practice, to a considerable extent, and with sufficient success to warrant me in recommending it as one of the most active, certain, and valuable of the emmenagogues. It may be used either in powder or decoction, though I greatly prefer the latter mode. My rule, in the admistration of the medicine, is to direct about four ounces of the decoction, more or less, during the day, according to the circumstances of the case. But at the time when the menstrual effort is expected to be made, and until the discharge is actually induced, I increase the dose as far as the stomach will allow, having given sometimes as much as two ounces every hour. In the interval of the menstrual periods, I lay aside the medicine for a week or two, as, without these intermissions, if it does not lose its power, it becomes disgusting to the patient."

Dr. Chapman directs the decoction to be made by putting one ounce of the bruised root in a pint of boiling water, in a covered vessel, and reducing it one-third by slowly simmering; and recommends that its nauseating tendency should be averted by the addition of an aromatic bitter. I have not found my patients able to bear so large doses as those indicated by Dr. C., and have been wont to add liquorice root, which disguises the peculiar taste of the senega, and to continue the process until it is reduced to one-half. A table-spoonful, three times daily, of this strength, is generally tolerated with difficulty. My habit is, when I can determine the period at which the natural tendency to the discharge will occur, to give the medicine in these doses for a fortnight before; and then, as Dr. C. advises, I have suspended it until the same period is again approaching. The causes of interruption to the menstrual discharge, being various, it is of course impossible to find any remedy which will meet every case. Where it depends on debility, or accompanies an anemic state of the system, other remedies than senega are more appropriate, or should be conjoined with it. Iron, aloes and myrrh, in combination, form an excellent remedy in such cases. The senega is appropriate to those cases where the suppression has been caused by improper exposure, and to those very frequent instances in which there is but little disturbance of the general health.

Every practitioner in our large cities must have had his attention arrested by the numerous calls for advice on account of obstruction, on the part of newly arrived immigrants, who complain of headache, and miserable general feelings, with swelling of their lower extremities. To what cause we are to ascribe the interruption of the natural functions, under such circumstances, it is difficult to say. The same result has been noticed in the cases of young women coming from the country to Paris. It is not, therefore, due to any impression made by the sea atmosphere, but, very probably, is caused in both cases by a less nutritious diet than has been customary, and the confinement in a vitiated atmosphere.

In those cases in which hemorrhoids, or an irritable condition of the lower bowels, prohibit the resort to the formulæ into which aloes so generally enter, the senega may be resorted to with benefit, and also, when there is a diseased state of the ovaries or uterus itself. I have not yet tried it in cases in dysmenorrhœa, with scanty secretion, but believe it will be found a very admirable remedy for these cases, which are so distressing to the habitual sufferer, and vexatious to the physician. I shall certainly take an early opportunity to test its powers, combined with some of the narcotic extracts. Hellebore and hyoscyamus have been the agents on which I have heretofore relied, with a good degree of satisfaction; and the senega appears to me to partake of the same character as the hellebore, without that tendency to purge which is often displayed by the hellebore when given in full doses. I am aware that some of our best teachers are disposed to deny the existence of a class of remedies having a specific tendency to promote the menstrual flow, and rely on general treatment for the restoration of this function when suspended. This is, perhaps, a natural reaction from the disposition to rely on specific remedies in all cases. Either extreme is unsound. We may not disregard the state of the general health, but must adapt our specific means to meet special indications. I know of no reason to doubt the tendency of certain remedies to produce an action of the uterus in its unimpregnated state, which would not lie with equal force against the action of calomel on the liver and salivary glands, or ergot on the same organ at the time of parturition.—*Med. Ex., and Rec. of Med. Sci.*

